# Sir Andrew Clark

**Published:** 1894-01-06

**Authors:** 


### SIR ANDREW CLARK.

Last year there died, too, a fellow-countryman of our own, who, in the greatness of his humanity, in his ardour for knowledge, and in his power of applying knowledge to the highest purposes of the physician's art, has seldom been equalled, and has probably never been surpassed. Sir Andrew Clark, who, like Charcot, died in his sixty-eighth year, was a rare man. If he had not been a rare man he would never have been Sir Andrew Clark. The outside world does not yet know, but we who are in actual medical practice know, what a concentration of capable intellect has swooped down upon the medical science and art during the last forty years. The man of merely ordinary mental powers is speedily submerged under all this variety and concentration of intellectual capacity. If in these circumstances a particular individual holds his own; if, still more, he emerges, slowly, perhaps, but surely and actually until he is seen even by the busy world to be conspicuous amongst, and distinctly above all his contemporaries, there is certainly something about that man which is not to be found in the average specimens of humanity. It is fit that Andrew Clark should be compared with a man like Charcot. Charcot contributed much to the science and literature of his profession. Clark contributed little. But he contributed to his profession what is quite as essential as the knowledge of additional facts, or the competent interpretation thereof. Clark did not devote his life to the mere accumulation of crude materials; he was the scientific and admirably cultured artist, who combined those materials to their highest capacity for the noble work of the alleviation of human suffering and the physical and mental advancement of mankind. The science which quarries is admittedly indispensable, but the science which builds is equally indispensable, and indeed constitutes the chief *raison d'être* of the quarrying. To say that a man like Sir Andrew Clark is not to be ranked with the first men of science of his time because he was a builder rather than a quarrier is to speak with the judg ment of a stupid child who has never been able to get beyond the mere A B C of the vast and infinitely diversified universe which it is the fortune of science to investigate.

